# Psychometric Properties of a Cyberaggression Measure in Mexican Students

**DOI:** 10.3390/bs14010019

**Published:** 2023-12-25

**Authors:** Christián Denisse Navarro-Rodríguez, Sheri Bauman, José Ángel Vera Noriega, Angel Emigdio Lagarda Lagarda

**Affiliations:** 1 Regional Development Department, Centro de Investigación en Alimentación y Desarrollo (CIAD), Hermosillo 83304, Mexico; christiandenisse.navarro@unison.mx (C.D.N.-R.); avera@ciad.mx (J.Á.V.N.); 2 College of Education, University of Arizona, Tucson, AZ 85721, USA; 3 Department of Psychology and Communication, Universidad de Sonora, Hermosillo 83000, Mexico

**Keywords:** cyberbullying, psychometrics, aggression, model validation, adolescents

## Abstract

Cyberaggression is an important problem today; it can affect adolescents in different ways. Therefore, reliable and valid measures are necessary to better study the phenomenon. The aim of the present study was to generate validity and reliability evidence for a Spanish-language cyberaggression scale from Garaigordobil, (2017) in a population of 1695 adolescents from northwestern Mexico (51.5% female) between 12- and 15-years-of-age. The results of this study contribute to the research and focus on cyberaggression in adolescents in Mexico. The measure used in this paper detects the different roles, including the bystander, rigorously testing the reliability and validity of the measure, providing a solid tool to evaluate cyberaggression in the Mexican context and guide evidence-based interventions and policies in educational settings.

## 1. Introduction

Throughout human history, aggression has existed in interpersonal relationships. However, as different changes in culture, society, and technology have occurred, aggressive behaviors have changed and adapted to their evolving context. Currently, much social interaction among young people occurs through social networks/cyberspace [1]. Information and Communication Technologies (ICTs) have given rise to new opportunities such as access to information, stimulating cross-cultural interaction, and improving communication and socialization. Despite that, cyberaggression issues such as cyberbullying, catfishing, scamming, cyber dating violence, sextortion, etc., have emerged [1]. It has become a global problem for adolescents in recent years, and scholars have devoted attention to this [2].

Researchers have used the terms “cyber victimization, e-bullying or electronic bullying, cyberstalking, electronic aggression, online harassment, cyber harassment, electronic victimization, peer victimization in cyberspace, cyber violence, online bullying, or ill-intended behaviors in cyberspace” interchangeably [3] (p. 3), and there is also no consensus on the definition, since some take into account aspects such as vulnerability or differences in power, repetition, and the intentionality of the act, while other studies do not [3]. For this reason, the term cyberaggression is used in this study, because it is an umbrella term that covers all cyber-based aggressive behaviors, defined as an act that seeks to cause harm through electronic means [4,5,6], even when there is no difference in power between the perpetrator and the victim and there is no repetition by the perpetrator. However, when referring to research, we retain the term used by the original authors.

Cyberaggression has been related to various negative outcomes in people who are involved in any role, but most of the studies of its consequences have focused on perpetrators and victims, even though it can affect bystanders too [7]. The negative effects can be physical, emotional, social, and psychological. Some of harmful outcomes are distress [8]; mental health problems such as anxiety, depression, fear [9], and low levels of self-esteem and empathy [10]; social exclusion [11]; and suicide attempts [12].

Findings regarding the prevalence of cyberaggression or cyberbullying are inconsistent, which may be due to measurement differences [3]. From ten studies carried out in Mexico, Vega-Cauich [13] determined that victimization prevalence rates ranged from 3% to 52%, while perpetration rates ranged from 3% to 23%. In addition to this, existing research in Mexico on the characterization of the three roles (aggressors, targets, and bystanders) is sparse. Therefore, the present study seeks to validate a Spanish-language scale that measures cyberaggression that can be used by Mexico and other Latin American countries, from the perspective of the participant roles [14] involved.

### 1.1. Cyberaggression in Mexico

Cyberaggression is recognized as a problem internationally, although most studies have been carried out in the United States, Europe, Australia, and to a lesser extent in other countries of the Global North, leaving research in the Global South lacking [10,15,16].

The National Survey on Availability and Use of Information Technologies in Households (ENDUTIH) reported that, in 2020, 84.1 million people in Mexico were internet users, which represents 72% of the population six years of age or more. Regarding location, 78.3% of the urban population are internet users, contrasting with 50.4% of the population in rural areas [17]. Seventy-six percent of the Mexican population aged six or older use a cell phone, 91.8% of which are smartphones. The most-used applications are instant messaging and tools for access to social networks.

The Module on Cyberbullying (MOCIBA), whose aim was to generate statistical information on the prevalence of the problem in people aged 12 and over who use the internet, in addition to characterizing the situations experienced, was administered in 2020 to a sample of 103.5 million Mexicans [18]. The results indicated that 75% of the sample stated that they had used the internet in the last three months; of the 75% who had used the internet, 21% declared that they had experienced some type of cyberaggression in the last 12 months. The distribution of cybervictimization was mainly concentrated in persons ages 12 to 19 years old, where 22.2% of males and 29.2% of females have been victims; those from 20 to 29 years old had very similar numbers (23.3% males and 29% females).

In this same study on cybervictimization in Mexico [18], males reported greater victimization in most situations but with only a small difference between genders. However, it is notable that females experienced a high prevalence of situations of a sexual nature, with differences of more than 15% between genders [18]. On the other hand, among the victims who claimed to know their aggressor, it was found that both men (59.4%) and women (53.2%) reported that most of the aggressors were males, while females were less frequently reported as aggressors (males 13.7% and females 18.6%).

Vega-Cauich [13] carried out a meta-analysis of bullying and cyberbullying, which synthesized the studies published in Mexico between 2009 and 2017. This researcher concluded that cybervictimization occurs in 21% of the student population between 10 and 22 years old and that cyberaggression is perpetrated by 11% of students. Other investigations carried out in the country reported rates of cybervictimization of between 2.4% and 44% [19,20,21].

### 1.2. Participant Roles in Cyberaggression

According to Salmivalli [22], there are several participant roles in bullying: There are the victims, who are those students who suffer aggression; on the other hand, the bullies are the perpetrators of violent conduct against other students; and finally, there are the bystanders, who are the witnesses of violent events. These roles have also been described in relation to cyberaggression.

Although there are different reasons why perpetrators commit aggressive behavior, the search for attention is very important [23,24]. Therefore, the role of bystanders is crucial in the phenomenon of aggression between peers, since by witnessing violent acts and not intervening they function as reinforcers of the behavior. Salmivalli et al. [14] classified the bystanders into four different types: (1) Assistants are those who help the aggressors; (2) Reinforcers are those who support the aggressor by encouraging them and laughing at the victim; (3) Defenders are those who intervene by helping victims or reporting incidents to a school authority; (4) Uninvolved are those who avoid aggressive events and do not act.

Most of the extant research on cyberaggression has focused on the victims and aggressors [7], but the current research considers it relevant to also characterize the bystanders, since they are also affected by the problem; in addition, the role of these adolescents can be crucial in stopping or encouraging aggressors [7].

### 1.3. Factors Related to Cyberaggression

Meta-analyses published in recent years [25,26] have shown that there is considerable variation in the prevalence of cyberaggression, whether as a victim or as a perpetrator, according to demographic and individual factors such as gender, race or ethnicity, sexuality, personality, and weight, among many others. These meta-analyses highlight that gender is relevant, but they point out that it is still not clear how this relationship works, since some studies point to females as the main victims and males as aggressors; others have shown opposite results. Finally, there are studies that indicate that there is no significant relationship. Chun et al. [3], in their review of cyberbullying measurements, mention that there is a need for measures that are sensitive to sex because, even though many studies have found differences between females and males, this aspect is not considered when creating or validating the measures, and this is necessary to understand the relative impact of cyberbullying.

Regarding age, it has been found that, even though cyberbullying can occur at an early age during primary education (under 12 years of age), its highest peak is in adolescence, declining between 17 and18 years and adulthood [26,27]. Likewise, factors like time spent online and presence on social media using different apps are important [27], but what is perhaps more interesting is the interaction among these factors and those mentioned before, such as presence in social media and gender, access to technology (time spend online) and age [16].

### 1.4. Cyberaggression/Cyberbullying Measurements

Self-report has been the most widely used technique to measure cyberbullying, and it has been shown that descriptive, analytical, and explanatory analyzes can be carried out using that method [28]. In addition, when aiming to make multidimensional conceptual models or study the prevalence from the different ways in which bullying occurs, using a multi-item scale is recommended [29].

In recent years, several studies where cyberbullying and cyberaggression measures are developed have been published. The most complete and current review to date is that carried out by Chun et al. [3], in which they analyzed measures published until May 2020. These previous studies have provided important contributions to knowledge of the cyberbullying phenomenon; however, it is important to examine some common limitations that are mentioned in this study.

The first limitation of previously published measures is the evident problem of the lack of agreement between research on conceptualization and operationalization, which can lead to confusion about what is measured and what is not, as well as conclusions about the relationships with other constructs that are not valid. Ansary [30] mentions that, in the face of this problem, some researchers have used global measures of cyberaggression as indicators of cyberbullying. However, this compromises the internal validity and distorts findings on the true prevalence values of both problems.

Another limitation Chun et al. [3] emphasize is that all the studies were conducted in developed countries. Knowing that the sociocultural context affects the responses and understanding of the interviewees, it should be considered when developing a scale. The review by Herrera-López et al. [15] indicates that the values of cyberbullying in developing countries are close to those reported in developed countries, but that the publications are very few and of low impact, which fuels a technological gap between countries.

Chun et al. [3] also mentioned that even though 17.2% of the studies claim not to have observed gender differences regarding the victimization or perpetration of cyberbullying, 42.2% of the studies did report this difference. Despite this, they did not find measures that are sensitive to the variable, even though the results suggest that the way in which male and female experience the problem is not necessarily the same, so the authors suggest that gender-sensitive cyberbullying measures are needed to reflect the reality of adolescents in a more reliable way.

Finally, a fourth limitation mentioned in the systematic review is that the studies do not follow a guide for the development of the scales; in addition, the vast majority do not report the necessary psychometric analysis and tend to underestimate the importance of validation. The Standards for Educational and Psychological Testing proposed by the American Educational Research Association (AERA), the American Psychological Association (APA), and the National Council on Measurement in Education (NCME) exist precisely to promote a systematization in the development of tests and give a basis for researchers to support the quality of the measures, so it is important that they be used as a reference framework to guarantee solid practices in the procedures of validity and reliability of the measures.

There are several features that researchers must consider when we seek to create or validate a measure. Morgado et al. [31] urge researchers to pay special attention to numbers. The first quantity to contemplate is the number of participants, since it is important that the studies are carried out with large and representative samples. The second quantity emphasized is the number of tests to be carried out, since it is important not only to check the statistical reliability but also to demonstrate the validity of the construct, an aspect that, according to Chun et al. [3], some studies overlook. The last important quantity to consider is the number of items, because, even though small scales require less time from the respondent, the reliability of the scale can be compromised; so, it is necessary to prioritize scales with enough items, which will allow reliability to remain within the acceptable range.

Translating and adapting a measure from one culture to another must be done through an appropriate methodology that guarantees the stability of its meaning and metric characteristics across cultures. Beaton et al. [32] and Ortiz-Gutierrez and Cruz-Avelar [33] propose processes of cross-cultural adaptation of measures. In these processes, the problems of cultural and language adaptation that arise when taking an instrument from one environment to another are reviewed.

Herdmann et al. [34] states that words can have different meanings from one culture to another or may not have an equivalent term in a culture; even when the language is the same, the meanings can be totally opposite due the sociocultural context. Therefore, it is important that developers of new measures modify the items that are not suitable at a conceptual level in the new context. Researchers always need to consider that the process of adapting and translating a measure to another culture does not guarantee that it will be valid in that new culture.

Because most of the studies use the term cyberbullying, in the present study, measures were sought that, even when this term was used, could be used to measure cyberaggression, such is the case of the Cyberbullying Test from Garaigordobil [35]. This scale demonstrates, through factor analysis, that it identifies cybervictims and cyberaggressors and can also identify those people who are part of both groups (cybervictim/cyberaggressors), and includes a dimension to detect cyberbystanders, a feature that few measures have.

Garaigordobil [35] reports acceptable reliability values of the three dimensions, cybervictimization with α = 0.82, cyberaggression with α = 0.91, and cyberbystander with α = 0.87. An EFA is presented as a test of internal validity and reported an explained 42.39% of the variances, with a three-factor structure. In addition, it demonstrates good convergent and divergent validity. The author also performed a confirmatory factor analysis that confirmed the fit of the model in the three factors with good statistical values (χ^2^/df = 4.88, CFI = 0.91, GFI = 0.92, RMSEA = 0.056, and SRMR = 0.050). This scale was administered to a representative sample of secondary and high school students in Spain, including adolescents between 12 and 18 years old, in an equitable sample with respect to gender.

By using the results without differentiating between the frequency of the responses, the Garaigordobil’s Cyberbullying Test is pertinent to this study; first, unlike other measures, its structure makes it possible to identify bystanders of cyberbullying, fundamental actors in the dynamic since their attitude can stop or encourage aggressors [7]. The second aspect is that their sample consisted of 3026 adolescents between the ages of 12 and 18 which means that they are similar to the target population of this research.

### 1.5. Present Study

Without a doubt, cyberaggression is an important problem today, it can affect adolescents in different ways regardless of the role they play, since it has been associated with mental health and other problems. In addition, its different roles are associated with different genders: aggressors are more often males, while victims, especially of sexual assaults, are often females, suggesting an urgent need for a gender sensitive measure and that the greatest participation in cyberaggression is during adolescence before the age of 17. In this sense, the research that is presented here aims to generate evidence of validity and reliability for a scale that measures cyberaggression, establishing its psychometric properties in a population of adolescent students in northwestern Mexico from the perspective of the participant roles involved. To achieve this aim in the most reliable way possible, the Standards for Educational and Psychological Testing proposed by AERA, APA, and NCME [36] were used as a framework for the psychometric analysis procedure.

## 2. Materials and Methods

### 2.1. Participants

A stratified random sampling was carried out among public schools, while community, private, and multigrade secondary schools were excluded. Ten percent of the schools from nine municipalities were chosen at random, in order to obtain greater variability and a representative sample for the state of Sonora in northwestern Mexico. The classrooms in each school were also randomly chosen, taking a group from the second grade and one from the third grade (equivalent to 8th and 9th grade in the American system). Students who, due to some physical or cognitive condition, could not answer were excluded from the sample.

The sample was made up of 1695 students, distributed among 55 schools. The second-grade sample was made up of 760 students (44.8%) and the third-grade sample comprised 935 students (55.2%). Age was not reported in the survey, but it is known that in these grades it ranges between 12 and 15 years; and, with respect to gender, equitable samples were obtained, with 873 female (51.5%) and 822 male (48.5%).

During data collection, survey administrators reviewed the response sheets to identify mischievous or inattentive response patterns to exclude such subjects. Subsequently, during data capture, those participants who had more than 10% of missing data were also excluded while responses were imputed with the mean of the item when participants had less than 10% of missing responses [37].

### 2.2. Procedure

In 2017, an agreement was established with the School Safety Management of the Ministry of Education and Culture (SEC) to study antisocial behavior in public elementary schools in the state of Sonora. A work team, made up of psychologists and psychology students, was trained to standardize the application procedure. The government agency notified the selected schools about the study by email and work teams of 2 to 5 people visited the schools.

The administration of all the measures took place in each school during class hours from October to November 2018. The trained staff presented themselves to the responsible management or teaching staff, who provided support with the selection of groups and location of the classrooms for survey administration. Within the classrooms, the students were asked to participate in the study, explaining that it was voluntary and confidential, they were instructed to provide their answers on the electronic sheets, and they were asked to sign an informed consent. The procedure performed complied with the Code of Ethics of the Psychologist of the Mexican Society of Psychology [38].

### 2.3. Measures

A sociodemographic questionnaire was included, with personal and academic data of the student, such as gender, grade, school performance, and social networks used, among other aspects.

Cyberaggression Measure: The Cyberbullying Test created by Garaigordobil [35] was modified to adapt the vocabulary to the population participating in the study. This measure includes 45 cyberaggression behaviors, grouped by the role that is enacted in the cyberaggression phenomenon. Each role is represented by a set of 15 items, exploring the experiences of the participant, depending on whether they were a victim (e.g., have you ever received offensive and insulting calls on your mobile or on the internet?), aggressor (e.g., have you ever made offensive and insulting calls using your mobile phone or the internet?), or bystander (e.g., have you ever seen offensive and insulting calls made via mobile or the internet?). Cronbach’s alpha = 0.91. These items focus on the behavior, regardless of the electronic means by which it was carried out. The responses were presented on a Likert-type scale with frequencies options as follows: never, sometimes, many times, and always.

A cultural adaptation of the Spanish measure was carried out considering linguistic and cultural factors. An iterative filtering procedure was carried out by a group of native researchers from the Northwest zone and from the state of Sonora who independently carried out their linguistic adjustment of the material in Spanish from Spain, taking care that the items conserved a semantic, conceptual, idiomatic, and experiential or cultural equivalence, adapting the terms to the Mexican culture (e.g., mobile modified to cell phone and hang it on internet changed to upload it to the internet).

Finally, it was reviewed by five specialists in social sciences who know the language and culture of young people between the ages of 12 and 15, following the recommendations of Beaton et al. [32] and Ortiz-Gutiérrez et al. [33], considering that this process of adaptation and translation of a measure to another culture does not guarantee that it is valid in that new culture.

Attachment to the Neighborhood Measure: Developed by Oliva et al. [39] focuses on feelings of belonging to the neighborhood. The scale consists of 6 items, evaluated on a Likert scale from 1 to 5, where 5 indicates the highest degree of agreement. The validity and reliability of this measure have been previously established in the Sonoran population by Vera et al. [40].

Bullying test: The measure created by Garaigordobil [41] evaluates 4 types of face-to-face harassment—physical, verbal, social, and psychological. This scale comprises 12 items, grouped into the roles of aggressor, victim, and bystander and evaluated on a frequency scale, with four points from never to always. The reliability and validity of the bullying test have been previously confirmed in the Sonoran population [42].

### 2.4. Data Analysis

Data were first subjected to Parallel Analyzes (PA) given the strong evidence that this is the most suitable method to determine the appropriate number of factors to retain [43].

Confirmatory Factor Analysis (CFA) was used to collect evidence about the structural validity based on the theoretical framework of the measure of the Cyberbullying Test [35], which has three subscales: victim, perpetrator, and bystander. To test the global fit, we used the following fit criteria of the Comparative Fit Index (CFI) and the Tucker–Lewis Index (TLI) higher than 0.9 and values of Root Mean Square Error of Approximation (RMSEA) and Standardized Root Mean Square Residual (SRMR) lower than 0.08 [44,45]. The loading of the first item of each factor was set to 1 to identify the model. The internal consistency was measured using Cronbach’s alpha.

Using the results obtained from the CFA, the Item Response Theory (IRT) approach was used with the Rasch model, using the Andrich Rating Scale Model (RSM) for polytomous items, to assess the fit of items to the model and confirm the unidimensionality of the subscales, based on infit and outfit MnSq with a range between 0.5 and 1.5; the difficulty of each item was also analyzed [46].

Correlations measured using Pearson’s r coefficient were used to assess convergent and discriminant validity of the measure, using the presence of correlation with a bullying measure as evidence of convergent validity and the absence of correlation with attachment to the neighborhood as evidence of discriminant validity.

As the last construct validity test, a factorial invariance across the gender samples was performed. Using the three-factor model (cyberaggressor, cybervictim, and cyberbystander), we computed a Multi-Group Confirmatory Factor Analysis (MGCFA) with males and females to addresses the configural invariance and measurement invariance of the model. The change in the value of CFI (∆CFI) and the change in the value of RMSEA (∆RMSEA) caused by invariance constraints were examined, considering that there should not be a ∆CFI ≤ −0.01 or a ∆RMSEA ≥ 0.015 [47].

PA and convergent and discriminant validity were analyzed using SPSS 26 [48]; internal consistency and Rasch models using WINSTEPS 3.65.0 [49]; and CFA and MGCFA using AMOS [50].

## 3. Results

In the PA, the random values generated by Monte Carlo simulations and the 95th percentile were used, which showed that the optimal number of factors is three (Table 1); so, the three subscales of the original measure were used in the calibration of items using the Rasch model procedure. In the first round of analysis, one item on each scale revealed an infit or outfit adjustment surpassing the established criteria, so three items were removed because they were inconsistent with the Rasch model (Table 2). The analyses were performed again for each scale where all item scores presented a satisfactory fit with their construct; values were between 0.75 and 1.42 as minimum and maximum values of infit and 0.58 and 1.44 as minimum and maximum values of outfit (Table 3).

Taking the results of the previous analysis as support, a CFA was performed using only the items that resulted in satisfactory values in the Rasch analysis. The initial model showed a poor fit (χ^2^(df) = 5876.518 (816), χ^2^/df = 7.202, TLI = 0.88, CFI = 0.89, RMSEA = 0.061 (90% CI = 0.059–0.062), and SRMR = 0.042). Cronbach’s alpha = 0.96. The modification indices were reviewed, then the covariances between some errors were allowed and two items from the dimension of cybervictimization, one item of cyberaggression, and one of cyberbystanders were eliminated (Table 2), resulting in a three-dimensional model with an acceptable fit (χ^2^(df) = 2965.71 (635), χ^2^/df = 4.670, TLI = 0.93, CFI= 0.94, RMSEA = 0.047 (90% CI = 0.045–0.048), and SRMR= 0.039). Cronbach’s alpha = 0.96.

Table 4 presents the descriptive statistics of the dimensions resulting from the cyberaggression measure. The highest mean was obtained by the cyberbystander dimension (M = 1.32, SD = 0.51) and the lowest by the cyberaggressor (M = 1.14, SD = 0.41). In the comparison between males and females, all the means are higher for males. The three values for the 95th percentiles that are between 2.00 and 2.54 indicate that most responses tend to deny having participated in the phenomenon of cyberaggression.

To measure convergent validity, each dimension was correlated with the dimensions of victim, aggressor, and bystander and significant correlations were obtained at the 0.01 level, and discriminant validity was determined in correlation with the measure of the Attachment to the Neighborhood; none of the three dimensions of cyberaggression obtained significant correlation results (Table 5).

Finally, the MGCFA was performed to test the measurement invariance of the cyberaggression scale. Initially, the configuration invariance model (M1) was tested, where factor loadings, intercepts, and error variances were allowed to be freely estimated. The indices obtained (CFI = 0.913; RMSEA = 0.041; and χ^2^/df = 3.806) indicated that the fit of the model to the data was adequate (Table 6).

In Model 2, the factor loadings were restricted to be equal between male and female, and the comparison with Model 1 in the ∆CFI was <0.01 and the ∆RMSEA was <0.015. When comparing Model 3—in which, in addition to the factor loadings, the intercepts between the groups were restricted—with Model 2, there were no significant changes between the CFI and the RMSEA. Finally, in Model 4, which is strict invariance, the error variances were also restricted, and in its comparison with Model 3 the ∆CFI > 0.01 and the ARMSEA > 0.015, contrary to expectations, resulting in a partial invariance but enough to perform analyzes of moderation effects between genders.

## 4. Discussion

The aim of the present study was to generate validity and reliability evidence for a cyberaggression scale in a population of adolescents from northwestern Mexico. The Garaigordobil Cyberbullying Test [35] was designed to obtain information on the different roles of cyberbullying in the Spanish population. However, even though the original measure was in Spanish, it was necessary to adapt the items to the context and terms used in Mexico so that Mexican adolescents had an adequate understanding of the measure.

The results of this study contribute to the research and focus on cyberaggression in adolescents in Mexico. The measure in this article differs from other measures by detecting the different roles, including the bystander, contributing to the state of knowledge about cyberaggression and ratifying the presence of this actor that has not been sufficiently explored in the cyber context [7]. This has implications in practice, since it is an adequate measure to evaluate cyberaggression in Mexican adolescents and facilitates the identification of aggressors, victims, and bystanders of cyberaggression and, thus, the planning of actions to address the problem.

The elimination of certain items in the measure of cyberaggression based on rigorous analysis results allowed us to obtain a precise and reliable measure of the phenomenon. By performing analyzes such as the Rasch and the confirmatory factor analysis, we can identify problems in the items of a scale and eliminate those that do not meet the necessary standards to guarantee its validity and reliability. The removal of items that do not work as they should, due to inconsistencies in responses, discrimination issues, social sensitivity, redundancy issues, or ambiguity, can significantly improve the quality of the measure and its ability to adequately capture the phenomenon it is intended to study. In addition, these analyzes not only make it possible to improve the quality of the scales but also help to guarantee the applicability of the results obtained in intervention and prevention contexts.

Each of the analyzes and indicators presented in this study are important since they represent a methodological contribution to the field of study of cyberaggression. Of these, the analysis of invariance by gender is noteworthy, which is added in this study to the analyzes previously carried out in the validation of Garaigordobil [35]. This analysis meets the need exposed by Chun et al. [3] of measures sensitive to this variable, allowing comparative analyzes to be made of these groups via the means of other factors [51]. Mexican researchers will be able to use this measure with confidence that it is adequate since the necessary psychometric tests have been carried out to support its validity and reliability. Currently, validity and reliability cannot be a property of the measures but rather represent the legitimacy of their use for specific objectives [36]. This metric is useful for researchers because it allows carrying out an orderly and systematic application of the theory, with reliable statistical models that allow precision and specificity in relation to the measurements. Having valid and reliable measures for populations with specific sociocultural characteristics contributes to the understanding of the phenomenon studied, since it makes it possible to analyze the individual differences of the subjects more precisely.

### Limitations and Recommendations

Our study has some limitations that must be acknowledged. Firstly, data collection was carried out through self-reports, which introduces the possibility of responses influenced by social desirability bias [52]. Another important aspect is that we used a cross-sectional design, which means that we collected data at a single point in time. Although this design is useful for studying certain phenomena and was useful in completing the aim of the present study, it has some limitations, particularly with respect to generalization. Specifically, it is possible that our findings may not fully represent all regions of the country. This could be due to differences in demographic, social, cultural, or economic factors across different regions, which may affect the prevalence, incidence, or severity of the phenomenon under investigation; cross-cultural studies are essential to assess the replicability of the measurement model in a culturally diverse population. Likewise, these results cannot be generalized to a non-student population.

Consequently, caution is advised when interpreting our results and generalizing them in other contexts. However, we strongly recommend that researchers in other regions replicate the validity procedure we carried out in this study, to advance our understanding of the phenomenon and inform evidence-based interventions and policies.

It is important to mention that the decision to apply the scale after cultural adaptation was based on the need to ensure cultural relevance and adequate understanding by the population studied. Nevertheless, we recognize that the lack of prior data in combination with the original scale may limit the ability to assess the specific impact of adaptations on responses. Future research could consider prior application of the original scale to gain a more complete perspective on the influence of cultural adaptations on outcomes.

## 5. Conclusions

As researchers, it is essential that we ensure the scales and measures we use are valid and reliable. Invalid or unreliable measures can lead to inaccurate or inconsistent findings, which can have serious implications for research and practice. Therefore, we believe it is crucial for researchers to rigorously test the validity of their measures before using them in their studies. By doing so, we can increase the confidence and trustworthiness of our findings and advance the scientific understanding of cyberaggression.

Our findings not only highlight the importance of accurate measurement but also offer practical insights for schools, suggesting that prevention and intervention strategies must consider the diversity of participants’ roles, presenting an approach to addressing cyberaggression in educational settings.

A notable characteristic of our study is that it incorporates Rasch analysis, increasing the methodological rigor of our scale. This addition contributes to the methodological diversity available to researchers, which strengthens the fundamental aspects when analyzing the psychometrics of the scales.

This meticulous process of analyzing the scale emphasizes its validity, which provides both researchers and professionals with a solid tool to evaluate cyberaggression in the Mexican context. Consequently, our research not only advocates for the importance of accurate measurement procedures but also provides tangible insights, which guide evidence-based interventions and policies in educational settings.

## Figures and Tables

**Table 1:** Actual and random eigenvalues for parallel analysis of the cyberaggression measure.

Actual Eigenvalue	Average Eigenvalues	95th Percentile Eigenvalue
18.712	1.301	1.328
3.429	1.271	1.292
2.803	1.248	1.266
1.217	1.229	1.247
1.037	1.212	1.225

**Table 2:** Table of items removal from Rasch and Confirmatory Factor Analysis (CFA).

Removed Item	Reason for Removal
Cybervictim	
- Have you been harassed to try to isolate yourself from your contacts on social networks?	Infit/outfit surpassed the established criteria
- Have you been sent offensive and insulting messages via cell phone or the internet?	Inappropriate covariances in the CFA
- Have someone blackmailed you, forcing you to do things you did not want in exchange for not divulging your private things online?	Inappropriate covariances in the CFA
Cyberaggressor	
- Have you sent offensive and insulting messages by cell phone or internet?	Infit/outfit surpassed the established criteria
- Have you blackmailed or forced someone to do things they did not want in exchange for not divulging their private things on the internet?	Inappropriate covariances in the CFA
Cyberbystander	
- Have you seen sending offensive and insulting messages via cell phone or over the internet?	Infit/outfit surpassed the established criteria
- Have you seen how someone has been blackmailed or forced to do things they did not want in exchange for not divulging their private things on the internet?	Inappropriate covariances in the CFA

**Table 3:** Rasch parameters of the measure.

	α	Infit		Outfit		Difficulty	
		Min	Max	Min	Max	Min	Max
Cybervictim							
Model 1 ^a^	0.93	0.67	1.46	0.49	1.48	−0.63	0.35
Model 2 ^b^	0.92	0.75	1.42	0.58	1.44	−0.60	0.36
Cyberaggressor							
Model 1 ^a^	0.95	0.84	1.60	0.50	1.57	−0.79	0.23
Model 2 ^b^	0.95	0.86	1.26	0.60	1.33	−0.25	0.19
Cyberbystander							
Model 1 ^a^	0.94	0.83	1.41	0.67	1.51	−0.33	0.39
Model 2 ^b^	0.93	0.84	1.30	0.69	1.21	−0.34	0.38

^a^ Model with all participants and all items. ^b^ Model without items with high infit/outfit values.

**Table 4:** Descriptive statistics of the cyberaggression dimensions.

	M	SD	Male		Female		Min	Max	Percentiles		
			M	SD	M	SD			25	75	95
Cybervictim	1.22	0.44	1.28	0.53	1.17	0.34	1.00	4.00	1.00	1.25	2.25
Cyberaggressor	1.14	0.41	1.22	0.50	1.07	0.28	1.00	4.00	1.00	1.08	2.00
Cyberbystander	1.32	0.51	1.38	0.58	1.25	0.42	1.00	4.00	1.00	1.38	2.54

**Table 5:** Convergent and discriminant validity.

	Victim of Bullying	Aggressor of Bullying	Bystander of Bullying	Attachment to the Neighborhood
Cybervictim	0.454 **	0.436 **	0.320 **	−0.016
Cyberaggressor	0.324 **	0.378 **	0.216 **	0.014
Cyberbystanders	0.327 **	0.337 **	0.313 **	−0.003

** Correlation is significant at the 0.01 level.

**Table 6:** Factorial Invariance.

	χ^2^(df)	χ^2^/df	CFI	RMSEA (IC 90%)	Contrast	∆χ^2^ *p* > 0.05	∆CFI ≤ 0.01	∆RMSEA ≤ 0.015
Model 1.	4833.124 (1270)	3.806	0.913	0.041 (0.039–0.042)				
Model 2.	5080.127 (1305)	3.893	0.908	0.041 (0.040–0.043)	M2 vs. M1	247.003 *	−0.005	0
Model 3.	5256.986 (1343)	3.914	0.904	0.041 (0.040–0.043)	M3 vs. M2	176.858 *	−0.004	0
Model 4.	11,017.737 (1414)	7.792	0.765	0.063 (0.062–0.064)	M4 vs. M3	5760.752 *	−0.139	0.022

Notes: Model 1 = configural invariance; Model 2 = M1 + weak measurement invariance; Model 3 = M2 + strong measurement invariance; and Model 4 = M3 + strict measurement invariance; * *p* < 0.0001.

## Data Availability

The data that support the outcomes of this study are available upon request from the corresponding authors.
